# Role of Toll-Like Receptor (TLR) 2 in Experimental *Bacillus cereus* Endophthalmitis

**DOI:** 10.1371/journal.pone.0028619

**Published:** 2011-12-06

**Authors:** Billy D. Novosad, Roger A. Astley, Michelle C. Callegan

**Affiliations:** 1 Department of Microbiology and Immunology, University of Oklahoma Health Sciences Center, Oklahoma City, Oklahoma, United States of America; 2 Department of Ophthalmology, University of Oklahoma Health Sciences Center, Oklahoma City, Oklahoma, United States of America; 3 Dean A. McGee Eye Institute, Oklahoma City, Oklahoma, United States of America; University Freiburg, Germany

## Abstract

*Bacillus cereus* causes a uniquely rapid and blinding intraocular infection, endophthalmitis. *B. cereus* replicates in the eye, synthesizes numerous toxins, and incites explosive intraocular inflammation. The mechanisms involved in the rapid and explosive intraocular immune response have not been addressed. Because Toll-like receptors (TLRs) are integral to the initial recognition of organisms during infection, we hypothesized that the uniquely explosive immune response observed during *B. cereus* endophthalmitis is directly influenced by the presence of TLR2, a known Gram-positive pathogen recognition receptor. To address this hypothesis, we compared the courses of experimental *B. cereus* endophthalmitis in wild type C57BL/6J mice to that of age-matched homozygous TLR2^-/-^ mice. Output parameters included analysis of bacterial growth, inflammatory cell (PMN) infiltration, cytokine/chemokine kinetics, retinal function testing, and histology, with N≥4 eyes/assay/time point/mouse strain. *B. cereus* grew at similar rates to10^8^ CFU/eye by 12 h, regardless of the mouse strain. Retinal function was preserved to a greater degree in infected TLR2^-/-^ eyes compared to that of infected wild type eyes, but infected eyes of both mouse strains lost significant function. Retinal architecture was preserved in infected TLR2^-/-^ eyes, with limited retinal and vitreal cellular infiltration compared to that of infected wild type eyes. Ocular myeloperoxidase activities corroborated these results. In general, TNFα, IFNγ, IL6, and KC were detected in greater concentrations in infected wild type eyes than in infected TLR2^-/-^ eyes. The absence of TLR2 resulted in decreased intraocular proinflammatory cytokine/chemokine levels and altered recruitment of inflammatory cells into the eye, resulting in less intraocular inflammation and preservation of retinal architecture, and a slightly greater degree of retinal function. These results demonstrate TLR2 is an important component of the initial ocular response to *B. cereus* endophthalmitis.

## Introduction

*Bacillus cereus* is a Gram-positive sporulating rod found throughout the environment. *B. cereus* is most commonly known as a food contaminant, causing self-limiting gastrointestinal symptoms such as vomiting or diarrhea. However, *B. cereus* can also cause severe infections, such as meningitis, food poisoning, bacteremia, and pneumonia. As one of the most feared ocular pathogens, *B. cereus* causes a uniquely rapid form of intraocular infection (endophthalmitis) that typically results in explosive intraocular inflammation, significant vision loss, and sometimes loss of the eye, within hours [1]. Its involvement in endophthalmitis typically occurs after open globe injuries. *B. cereus* has been isolated in as many as 46% of reported cases of post-traumatic endophthalmitis [2]. Post-traumatic endophthalmitis cases caused by *B. cereus* resulted in less than 30% of patients retaining useful vision, while only 9% of infected patients retained 20/70 vision or better. Nearly 50% of *B. cereus* endophthalmitis cases require enucleation of the eye [2].

During *B. cereus* endophthalmitis, loss of vision or the eye itself can occur despite proper and aggressive therapeutic intervention that may otherwise cure infection caused by other ocular pathogens such as *Staphylococcus aureus* or *Streptococcus pneumoniae* [1]. *B. cereus* endophthalmitis typically results in involvement of both the anterior and posterior segments, leading to inflammation of the vitreous (vitritis), aqueous humor, and cornea, with a hallmark corneal ring abscess. Rapid vision loss and severe ocular pain occurs, with systemic symptoms including fever and an elevated leukocyte count [1]. During the early stages of *B. cereus* endophthalmitis, the eye mounts an aggressive inflammatory response in an effort to eradicate intraocular organisms. Previous studies have demonstrated that metabolically inactive *B. cereus* triggers the explosive intraocular inflammatory response [3], suggesting that cell wall components play a role in inciting inflammation. *B. cereus* also synthesizes multiple toxins in the eye during infection [4], which are likely responsible for the rapid loss of vision that is a trademark of this disease [5]–[8]. The mechanisms underlying the inflammatory response and vision loss during *B. cereus* endophthalmitis remain an open question.

Initial recognition of bacteria during the acute stage of infection is critical in mounting an effective immune response. Bacteria are recognized by pattern recognition receptors known as Toll-like receptors (TLRs), which signal through the NF-κB pathway and upregulate the synthesis of cytokines and chemokines responsible for recruiting immune cells to the site of infection [9], [10]. TLRs have been identified in many cells throughout the eye, including retinal pigment epithelial cells, astrocytes, corneal epithelium, iris epithelium, and Muller cells [11], [12]. Studies have demonstrated the importance of TLR-mediated recognition of ocular pathogens during bacterial keratitis [13]–[17], but analysis of the role of TLRs in modulating posterior segment inflammation during bacterial infection is lacking.

*Bacillus* possesses known ligands for recognition via TLR2 such as peptidoglycan and lipotechoic acid. *Bacillus anthracis* has been shown to stimulate signaling through TLR2 [18]. Genetically, *B. anthracis* and *B. cereus* are close relatives and are considered monophyletic clones [19]. With the exception of the anthrax toxin, *B. anthracis* and *B. cereus* synthesize a similar complement of toxins and enzymes. Therefore, the similarities of *B. cereus* and *B. anthracis* may also extend to that of the cell wall, and their ligands may be recognized by TLRs in a similar fashion.

Because TLRs are integral to the initial recognition of organisms during infection, we hypothesized that the uniquely explosive immune response observed during *B. cereus* endophthalmitis is directly influenced by TLR2. We tested this hypothesis by comparing the pathogenesis of experimental *B. cereus* endophthalmitis in TLR2-deficient mice with that of infection in wild type mice.

## Results

### TLR2 expression in the retina during *B. cereus* endophthalmitis

**Figure 1:**
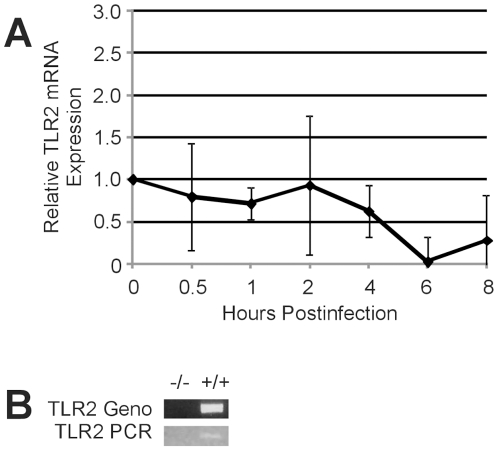
TLR2 expression in the retina during *B. cereus* endophthalmitis. C57BL/6J mouse eyes were injected with 100 CFU *B. cereus* and retinas were harvested at 0, 0.5, 1, 2, 4, and 8 h postinfection. (A) No significant change in retinal TLR2 mRNA expression was detected during infection. Values are mean ± SD of N≥4 retinas per time point (P≤0.05, 0 h postinfection compared with all other time points). (B) TLR2 PCR of wild type and TLR2^-/-^ strains used in this study. Reactions using primers for genotyping [41] or real-time PCR (Methods) are shown.

Studies have reported increased expression of TLR2 in response to infection [20]–[22] and inflammation [23]–[25], while others have reported decreased or attenuated expression [26]–[28]. Numerous *in vitro* studies document the activation of TLR2 by Gram-positive ligands, but these models may not represent the *in vivo* environment during infection. We therefore analyzed the expression of TLR2 in retinas of wild type mice during the course of infection (Figure 1). Quantitative real-time PCR of TLR2 mRNA in retinas demonstrated no change in expression during infection.

### Effect of TLR2 deficiency on intraocular growth of *B. cereus*

**Figure 2:**
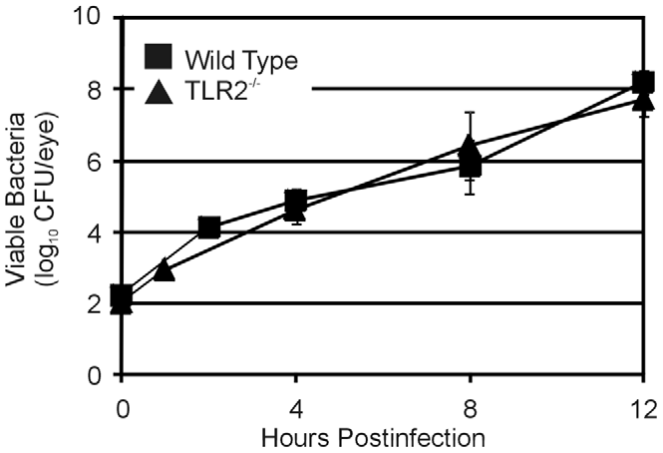
Bacterial growth during experimental *B. cereus* endophthalmitis. C57BL/6J wild type and TLR2^-/-^ mouse eyes were injected with 100 CFU *B. cereus*. Eyes were harvested, homogenized, and analyzed for bacterial growth. *B. cereus* grew to similar concentrations in infected eyes of TLR2^-/-^ mice and wild type mice (P≥0.05 at all time points). Values represent the mean±SEM of N≥8 eyes per time point for at least 2 separate experiments.

The intraocular growth rates of *B. cereus* were analyzed in infected eyes of wild type and TLR2^-/-^ mice (Figure 2). The growth rates of *B. cereus* in TLR2^-/-^ eyes were similar to that of wild type eyes when compared at 4, 8 and 12 h postinfection (P≥0.162). Growth rates reached 10^8^ CFU/eye by 12 h in wild type and TLR2^-/-^ mice. Ramadan *et al.* [29] previously showed that *B. cereus* grew to similar numbers in eyes of wild type mice of a similar background. This result demonstrates that the TLR2 functional deficiency does not affect the intraocular growth rates of *B. cereus* during endophthalmitis.

### Effect of TLR2 deficiency on retinal function during *B. cereus* endophthalmitis

**Figure 3:**
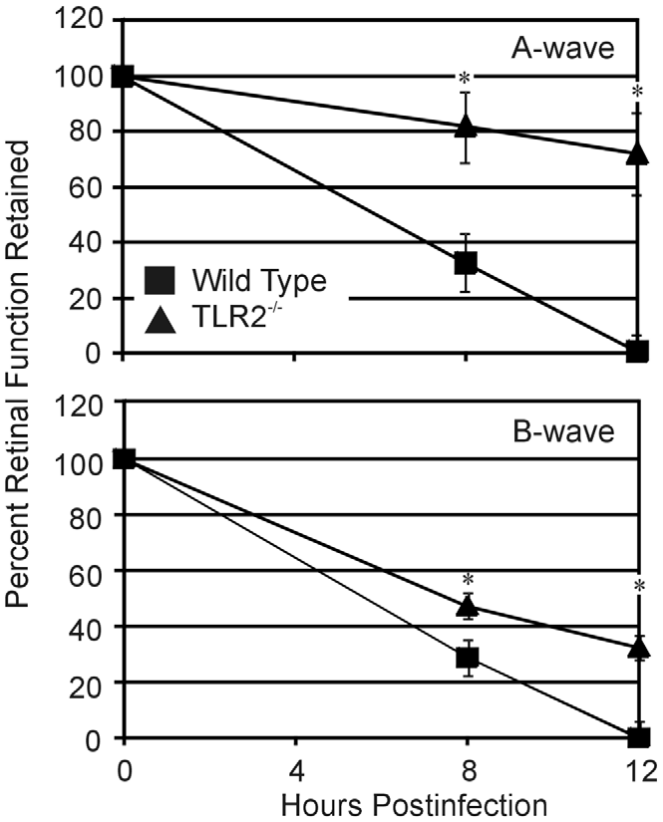
Retinal function analysis during *B. cereus* endophthalmitis. C57BL/6J wild type and TLR2^-/-^ mouse eyes were injected with 100 CFU *B. cereus*. Retinal function was assessed by electroretinography. At 8 and 12 h postinfection, A- and B-wave amplitudes retained were significantly lower in infected wild type eyes than in infected TLR2^-/-^ eyes. By 12 h, retinal function was abolished in infected eyes of wild type mice, and significant function loss was seen in infected eyes of TLR2^-/-^. Values represent the mean±SEM of N = 8 eyes per time point for at least 2 separate experiments. *P≤0.05.

Analysis of retinal function loss during infection is summarized in Figure 3. We observed significantly slower retinal function declines in infected TLR2^-/-^ eyes compared to that of infected wild type eyes. Reductions in A-wave function in TLR2^-/-^ eyes were significantly less than that of wild type eyes at 8 and 12 h (P≤0.004). The loss of B-wave function in infected eyes of TLR2^-/-^ mice was also significantly less than that of infected eyes of wild type mice at 8 and 12 h (P≤0.023). However, retinal function loss was considerable regardless of the TLR2 background of the infected eyes. These results suggest that while the TLR2 functional deficiency altered the speed at which retinal function was lost, the absence of a functional receptor ultimately had a minimal effect on overall vision loss in this model.

### Effect of TLR2 deficiency on intraocular inflammation during *B. cereus* endophthalmitis

**Figure 4:**
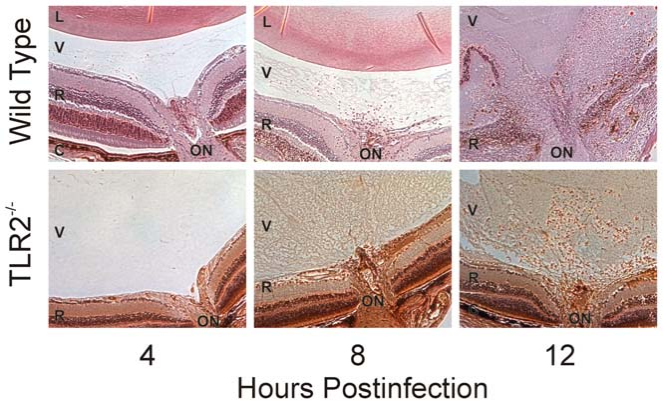
Retinal histology of *B. cereus* endophthalmitis. Wild type and TLR2^-/-^ mouse eyes were injected with 100 CFU *B. cereus*. Eyes were harvested and processed for hematoxylin and eosin staining. Infected TLR2^-/-^ had significantly less inflammation than infected eyes of wild type mice. Sections are representative of 4 eyes per group. L, lens; V, vitreous; R, retina; C, choroid; ON, optic nerve head. Magnification, 40X.

**Figure 5:**
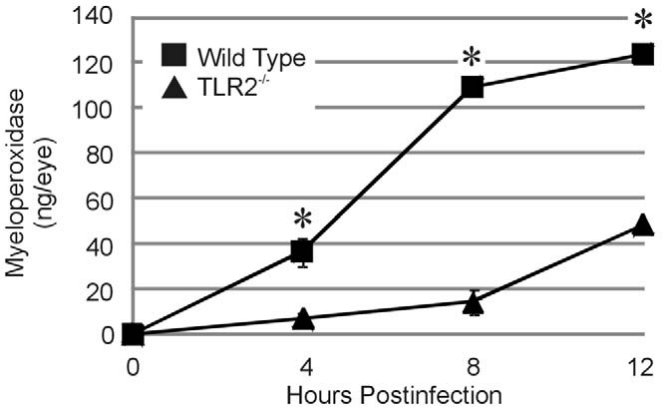
Infiltration of PMN into mouse eyes during *B. cereus* endophthalmitis. C57BL/6J wild type and TLR2^-/-^ mouse eyes were injected with 100 CFU *B. cereus*. PMN infiltration was estimated by quantifying MPO in whole eyes by sandwich ELISA. MPO concentrations were significantly higher in infected wild type eyes than in infected TLR2^-/-^ eyes (*P≤0.05), suggesting greater numbers of PMN in infected wild type eyes than in infected TLR2^-/-^ eyes. Values represent the mean±SEM for N≥4 per group for at least 2 separate experiments.

Figure 4 depicts a histological comparison of posterior segment inflammation in wild type and TLR2^-/-^ mice during *B. cereus* endophthalmitis. The images are centered at the optic nerve/retinal interface and include the vitreous. At 4 h, fibrin infiltrate and transient PMN were seen in the vitreous of infected wild type eyes. At the same time, the vitreous of eyes of TLR2^-/-^ mice were relatively clear and similar to that of mock-injected control eyes. At 8 h, PMN were seen in close proximity to the optic nerve head and significant fibrin infiltrate was observed throughout the vitreous in infected wild type eyes. Comparatively, there were very few PMN visible in the posterior segment of infected TLR2^-/-^ mice. At this time, significant fibrin infiltrate was observed in the vitreous of infected TLR2^-/-^ eyes, and retinas appeared to be intact. At 12 h, retinas of wild type mice were significantly disrupted and retinal layers were indistinguishable. Significant infiltration of PMN, fibrin infiltrate, and complete loss of retinal architecture was observed in infected wild type eyes at 12 h. At the same time, eyes of TLR2^-/-^ mice demonstrated minimal disruption of retinal structure and infiltration of PMN into the vitreous from the optic nerve, similar to that seen in wild type eyes at 8 h. These results suggested that retinal disruption and PMN influx into the posterior segment was delayed in eyes of TLR2^-/-^ mice. Myeloperoxidase (MPO) activities of infiltrating PMN are summarized in Figure 5. Significantly greater MPO activity was detected in infected eyes of wild type mice compared to that of infected eyes of TLR2^-/-^ mice at 4, 8, and 12 h (P≤0.0001). Similar levels of MPO were detected in infected TLR2^-/-^ eyes at 12 h and wild type eyes at 4 h (P = 0.0528). These data correlated with the histology data, further demonstrating that the delay in posterior segment inflammation resulted from the absence of functional TLR2.

### Effect of TLR2 deficiency on proinflammatory cytokines/chemokines during *B. cereus* endophthalmitis

**Figure 6:**
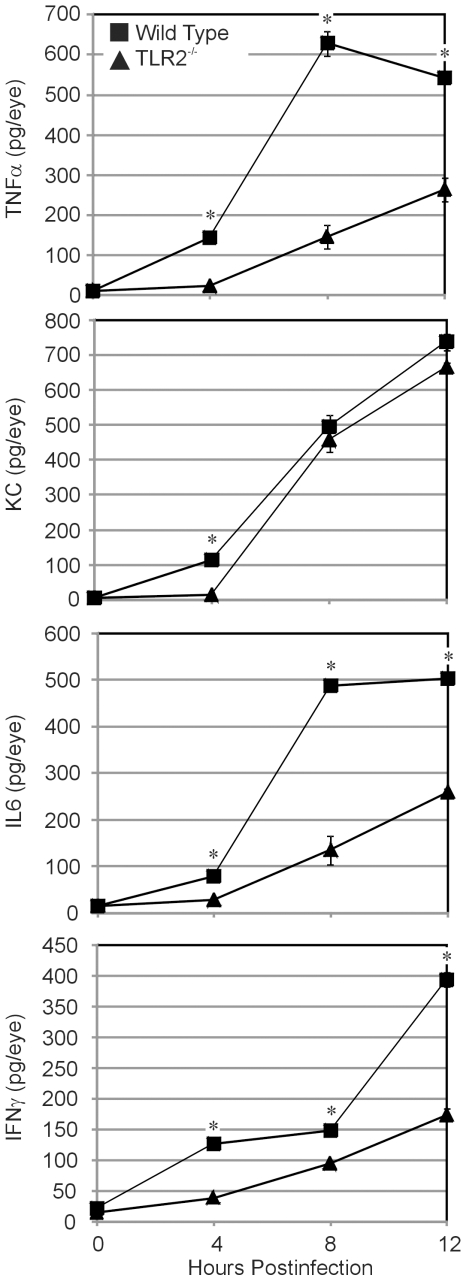
Proinflamatory cytokine and chemokine expression during experimental *B. cereus* endophthalmitis. C57BL/6J wild type and TLR2^-/-^ mouse eyes were injected with 100 CFU *B. cereus.* Ocular proinflammatory cytokines and chemokines were analyzed by sandwich ELISA. Overall, greater levels of TNFα, KC, IL-6, and INFγ were synthesized in infected eyes of wild type mice compared with that of infected eyes of TLR2^-/-^ mice. Values represent the mean±SEM for N≥6 per group for at least 2 separate experiments.

Levels of cytokines and chemokines in *B. cereus-*infected eyes are summarized in Figure 6. Infected TLR2^-/-^ eyes had significantly less TNFα,IL6, and IFNγ at 4, 8, and 12 h postinfection as compared to infected wild type eyes (P≤0.0005). KC values were also greater in infected wild type eyes compared to that of infected TLR2^-/-^ eyes at 4 h only (P≤0.0001). These results, together with the histology and myeloperoxidase activity data, confirmed the diminished intraocular inflammation in infected TLR2^-/-^ eyes during experimental *B. cereus* endophthalmitis.

## Discussion

The innate immune response is the first line of defense against an invading pathogen such as *B. cereus* [9], [10], [30]. Without that initial line of defense, especially in an immune-privileged environment such as the eye, pathogens would freely replicate, produce toxic factors, and damage tissue. Retinal tissue damage is irreversible, resulting in vision loss. This study unambiguously demonstrated that during *B. cereus* endophthalmitis, TLR2 directly influenced the severity of intraocular inflammation. The absence of a functional TLR2, shown in several other models to be essential for recognition of Gram-positive pathogens, altered the expression of proinflammatory cytokines and chemokines, resulting in delayed recruitment of PMN into the eye. This effect significantly limited intraocular inflammation, but had only a minimal effect on loss of retinal function during infection.

The absence of a functional TLR2 did not affect the growth of *B. cereus* in the intraocular environment. We previously reported that intraocular inflammation in TNFα-deficient mice was limited, resulting in a larger bacterial load and faster retinal function loss during endophthalmitis [29]. In the present study, intraocular bacterial loads were similar regardless of the presence of a functional TLR2, and despite the relative lack of inflammation that would otherwise serve to limit bacterial growth. These results suggest that the limited inflammation observed in TLR2^-/-^ eyes may have been sufficient enough to arrest the degree of uninhibited growth seen in TNFα-deficient eyes. It appears that a threshold of inflammation must be present in the eye to control bacterial growth.

Similar bacterial growth rates in eyes of wild type and TLR2^-/-^ mice may also indicate that similar levels of toxins were produced in these eyes. Throughout the course of *B. cereus* endophthalmitis, several toxins are produced, including hemolysins, phospholipases, enterotoxins and proteases [4]. Yet, there was a delay in retinal function loss in TLR2^-/-^ eyes and eventually, TLR2^-/-^ eyes lost significant function. Since intraocular bacterial growth (and presumably toxin production) was similar regardless of TLR2 genetic background, the differences observed in function loss may be attributed to differences in intraocular inflammation. We have previously shown that metabolically inactive *B. cereus* causes an inflammatory influx resulting in some retinal function loss [3]. Inflammation may therefore contribute to some aspect of retinal function loss, potentially disrupting the biochemical processes associated with the phototransduction cascade as inflammatory cells enter the retina or as retinal or inflammatory cells synthesize cytokines/chemokines that affect these processes. The roles of specific *B. cereus* cell wall components in the explosive intraocular response are being investigated.

The primary difference observed between endophthalmitis in wild type versus TLR2^-/-^ mice was the lack of significant posterior segment inflammation. In eyes of TLR2^-/-^ mice, proinflammatory cytokine/chemokine synthesis was arrested, resulting in delayed recruitment of PMN into the retina and vitreous. This limited inflammation was similar to that observed in this infection model in TNFα^-/-^ mice. When subjected to similar intravitreal challenge with *B. cereus*, eyes of TNFα^-/-^ mice had significantly less MPO and proinflammatory cytokines during the course of infection compared to that of eyes of wild type mice [29]. Retinal histology also showed preserved retinal architecture in TLR2^-/-^ mice compared to that of wild type mice. The lack of PMN recruitment in both TNFα^-/-^ and TLR2^-/-^ mice suggest that the lack of TNFα affected the recruitment of PMN into the eye in this model. The contribution of other important proinflammatory mediators to intraocular inflammation are being investigated.

Kumar *et al.* analyzed the efficacy of the synthetic TLR2 ligand, Pam3Cys, in altering the outcome of *S. aureus* endophthalmitis [31]. In that study, upregulation of TLR2 by Pam3Cys prior to infection led to reduced numbers of intraocular staphylococci and preservation of retinal function, compared to untreated infected control mice. Retinal TLR2 was upregulated upon intravitreal challenge with Pam3Cys, but downregulated upon intravitreal challenge with *S. aureus*. From this data, it is not clear at what time during experimental *S. aureus* infection TLR2 initiated the immune response. However, pre-infection challenge with Pam3Cys clearly altered the TLR2 response to *S. aureus* intraocular challenge, diminishing inflammation, reducing bacterial load, and preserving retinal function in this model. In our study, absence of TLR2 led to decreased inflammation, but no decrease in bacterial load and significant retinal function loss. Taken together, these results suggest that the mechanisms of the intraocular TLR2-mediated immune response to *B. cereus* and *S. aureus* are quite different. We [3] reported that the kinetics and degrees of intraocular inflammation caused by *B. cereus*, *S. aureus*, and *E. faecalis* were highly variable, suggesting that the intraocular immune response is organism-dependent. This is clearly the case with relatively avirulent organisms such as *S. epidermidis*, which causes infection and inflammation in the eye only at high concentrations [32], [33]. Suggested development of therapeutics that target pattern recognition receptors must therefore not only account for organism-dependent differences in recognition, but also realistic timing of administration for different infection scenarios.

In this study, retinal TLR2 mRNA expression did not increase during infection. Downregulation of TLR2 in response to infection has been reported during experimental *S. aureus* endophthalmitis [31]. Hyporesponsiveness to lipoteichoic acid has been suggested as a potential mechanism of limiting inflammation-induced damage [34]–[37]. Although TLR2 appears to be essential for initial bacterial recognition and rapid inflammation during *B. cereus* endophthalmitis, upregulation of TLR2 may not be necessary once the organisms are recognized and the inflammatory cascade has begun. Early mediation of the TLR2 pathway activated at the time of infection may potentially limit TLR2 signaling, unnecessary inflammation, and further damage. The fact that intraocular inflammation, although delayed, occurs at all in the absence of TLR2 suggests that pathogen recognition and the resulting response occurs by a redundant mechanism. As indicated earlier, other TLRs have been detected in cells throughout the eye [11], [12] and have been shown to be important in ocular infection [13]–[17]. We also found diminished inflammation in *B. cereus-*infected eyes of TLR4^-/-^ mice, results similar to that observed in infected TLR2^-/-^ eyes (Novosad and Callegan, unpublished work). *B. cereus* does not possess the classic TLR4 ligand, LPS. *B. cereus* does, however, secrete cereolysin O, a cholesterol-dependent cytolysin (CDC) similar in sequence and structure to that of other CDCs demonstrated to interact with TLR4 [38]–[40]. If both TLR2 and TLR4 respond similarly in the eye to infection with *B. cereus*, this may account for the unusually robust inflammation observed during this disease. Current studies are analyzing TLR4-*B. cereus* interactions in the eye and the triggers involved in these interactions.

The present results unequivocally demonstrated that a lack of functional TLR2 significantly altered the intraocular inflammatory response to *B. cereus* endophthalmitis. Because *B. cereus* is a rapidly blinding infection, therapeutics designed to delay its pathogenicity could prove to be an invaluable tool. Further understanding of the mechanisms by which TLR2 and other components of innate immunity respond to *B. cereus* and other organisms in the eye could facilitate the development of new therapeutic regimens to hamper inflammation and prevent vision loss during this blinding infection.

## Materials and Methods

### Experimental *Bacillus cereus* Endophthalmitis

Animals were used following institutional guidelines and the ARVO Statement for the Use of Animals in Ophthalmic and Vision Research. Wild type C57BL/6J mice (male 6–8 weeks of age; Jackson Laboratories, Bar Harbor, ME) and homozygous TLR 2^-/-^ mice [41] were used for infection studies. These mice were backcrossed onto a C57BL/6J background for 5–8 generations. Genotypes were verified using primer sets and PCR conditions developed for these knockouts [41]. Each mouse was anesthetized with a combination of ketamine (85 mg/kg body weight; Bionichepharma, LLC., St. Lake Forrest, IL) and xylazine (14 mg/kg body weight; Rompun; Bayer Corp., Shawnee Mission, KS). Each eye was topically anesthetized prior to intravitreal injections and analysis of retinal function by electroretinography (0.5% proparacaine HCl; Ophthetic; Allergan, Hormigueros, Puerto Rico). Experimental endophthalmitis was induced in mice as previously described with *B. cereus* strain ATCC 14579 (American Type Culture Collection [ATCC], Manassas, VA) [7], [8], [29]. Briefly, 0.5 µL of brain heart infusion media containing approximately 100 colony forming units (CFU) of *B. cereus* were injected into the midvitreous. The contralateral eye was not injected (absolute control).

### Electroretinography (ERG)

Electroretinography (ERG) is used to analyze retinal function and physiology. The retinal response is stimulated by a transient flash of light and results from a chain of electrical responses in the form of graded potentials evoked in each layer of the retina. The response consists of an A-wave measured in a negative amplitude below zero, followed by a B-wave measured from the trough of the A-wave to the highest positive value above zero. The leading edge of the A-wave provides a direct measure of photoreceptor activity, while the B-wave represents the action of Muller cells, bipolar cells, and second order neurons (amacrine and ganglion cells). Scotopic A- and B-wave amplitudes were recorded for each experimental (infected) eye compared with its fellow control (uninfected) eye (UTAS3000; LKC Technologies, Inc., Gaithersburg, MD). The percentage of retinal function retained was calculated as follows 100 – {[1 – (experimental A-wave amplitude/control A-wave amplitude)] x 100} or 100 – {[1 – (experimental B-wave amplitude/control B-wave amplitude)] x100} [7], [8], [29]. Scotopic ERGs were performed at 8 and 12 h postinfection (N = 8 eyes per group per time point, mean±standard error of the mean [SEM]). Scotopic ERGs were not performed earlier than 8 h because of the extended dark adaptation time needed for the mouse retina.

### Histology

Whole eyes were harvested at 0, 4, 8, or 12 h postinfection and incubated in an 85/15 paraformaldehyde/alcohol fixative for 24 h at room temperature. Whole eyes were then exchanged into 70% ethanol for 24 h and then embedded in paraffin. Sections were deparaffinized and stained in Harris hematoxylin solution for 8 min, counterstained in eosin-phloxine B solution for 30 sec, dehydrated through two changes of 95% alcohol and cleared in two changes of xylene. Images are representative of 4 eyes per group at each time point.

### Bacterial Quantitation

Bacteria were quantified in whole eyes as previously described [7], [8], [29]. Briefly, whole eyes were homogenized with 1-mm sterile glass beads (BioSpec Products, Inc., Bartlesville, OK) in 400 µL PBS and then 10-fold track diluted onto BHI agar. Values represent the mean±SEM for N≥8 eyes per time point.

### Myeloperoxidase (MPO)

Polymorphonuclear leukocytes (PMN) contain myeloperoxidase, a peroxidase enzyme stored in azurophilic granules. Previous studies have shown that PMN are the primary cell type entering the eye during the first 12 h of *B. cereus* endophthalmitis [8]. Estimating PMN influx into the eye was achieved by quantifying myeloperoxidase in whole eyes by sandwich ELISA (Mouse MPO ELISA Test Kit; Cell Sciences, Canton, MA), as previously described [29]. Negative controls included noninfected eyes. Eyes were analyzed for MPO activity at 4, 8, or 12 h postinfection. Results are reported as MPO ng/eye±SEM for N≥4 eyes per group per time point.

### Quantitation of TLR2 in Retinas

Eyes were collected and retinas dissected from each eye cup for quantitation of TLR2 by real-time PCR and Western blot. One retina was used for each real-time PCR reaction, with N≥4 retinas analyzed per group in duplicate. Primer efficiencies were verified by performing real time RT-PCR on a standard curve created with cDNA produced from qPCR total reference RNA (BioRad, Hercules CA). All primers were designed using Ensembl and Primer 3. Primers used for TLR2 were forward 5'-ATGCTTCGTTGTTCCCTGTGTTGC-3’ and reverse 5’-AACAAAGTGGTTGTCGCCTGCTTC-3'. The 2^−ΔΔCT^ standard curve method was used to evaluate the relative expression level of TLR2 in infected eyes of wild type mice. Quantitative real-time PCR was performed (iCycler iQ, BioRad) according to the manufacturer’s instructions. Briefly, the thermal cycling conditions were 40 cycles of 55°C for 30 sec and 95°C for 1 min. The cycle threshold (C_T_) was set for the target gene, where all amplicons were in the exponential phase of amplification. Data were analyzed using the relative standard curve method. All target C_T_ values reported by the iCycler software were normalized to the endogenous control, β-actin (target mean input – endogenous control mean input  =  target _N_). The resulting C_T_ value was then normalized to the untreated wild type control (target _N_/ control _N_ =  relative fold difference in target expression). A greater than 2-fold change in mRNA expression was considered significant.

### Quantitation of Cytokines and Chemokines

Ocular proinflammatory cytokines and chemokines were quantified as previously described [7], [8], [30]. Harvested eyes were mixed with a protease inhibitor cocktail (Triton X-100, 0.5 M EDTA, 10 mM sodium orthovanadate [Sigma] and Protease Inhibitor [Calbiochem, La Jolla, CA] in PBS, pH 7.4) and homogenized with glass beads. Commercial ELISA kits (Quantikine; R&D Systems, Minneapolis, MN) were used to analyze the levels of KC (IL-8), TNFα, IL-6, and IFNγ in accordance with the manufacturer’s instructions. Eyes were analyzed at 4, 8, or 12 h postinfection. Cytokine and chemokine concentrations were interpolated from standard curves. The lower limits of detection for each assay are as follows: KC, 2 pg/ml; TNFα, 5 pg/ml; IL6, 2 pg/ml; IFNγ, 2 pg/ml. Values are expressed as mean±SEM for N≥6 eyes per time point.

### Statistics

If not stated otherwise, results were the arithmetic means±standard errors of the mean (SEM) of all of the samples in the same experimental group. A two-tailed Student *t* test was used to determine the statistical significance of the data. Wilcoxon’s rank sum test was used for statistical comparison between groups. Statistical significance was determined at *P* < 0.05.
